# Power-Law Inter-Spike Interval Distributions Infer a Conditional Maximization of Entropy in Cortical Neurons

**DOI:** 10.1371/journal.pcbi.1002461

**Published:** 2012-04-12

**Authors:** Yasuhiro Tsubo, Yoshikazu Isomura, Tomoki Fukai

**Affiliations:** 1 Laboratory for Neural Circuit Theory, RIKEN Brain Science Institute, Wako, Saitama, Japan; 2 Brain Science Institute, Tamagawa University, Machida, Tokyo, Japan; 3 CREST, JST, 4-1-8 Honcho, Kawaguchi, Saitama, Japan; Gatsby Computational Neuroscience Unit, University College London, United Kingdom

## Abstract

The brain is considered to use a relatively small amount of energy for its efficient information processing. Under a severe restriction on the energy consumption, the maximization of mutual information (MMI), which is adequate for designing artificial processing machines, may not suit for the brain. The MMI attempts to send information as accurate as possible and this usually requires a sufficient energy supply for establishing clearly discretized communication bands. Here, we derive an alternative hypothesis for neural code from the neuronal activities recorded juxtacellularly in the sensorimotor cortex of behaving rats. Our hypothesis states that *in vivo* cortical neurons maximize the entropy of neuronal firing under two constraints, one limiting the energy consumption (as assumed previously) and one restricting the uncertainty in output spike sequences at given firing rate. Thus, the conditional maximization of firing-rate entropy (CMFE) solves a tradeoff between the energy cost and noise in neuronal response. In short, the CMFE sends a rich variety of information through broader communication bands (i.e., widely distributed firing rates) at the cost of accuracy. We demonstrate that the CMFE is reflected in the long-tailed, typically power law, distributions of inter-spike intervals obtained for the majority of recorded neurons. In other words, the power-law tails are more consistent with the CMFE rather than the MMI. Thus, we propose the mathematical principle by which cortical neurons may represent information about synaptic input into their output spike trains.

## Author Summary

The brain is a highly noisy information machine, making a striking contrast with man-made electric computers to which noise is merely harmful. However, little is known about the way neurons process information in the noisy states. Here, we explore the principle of noisy neural information processing in accurately recorded spike trains of *in vivo* cortical neurons. We found that their irregular spiking exhibits power-law statistics of inter-spike intervals. While the power law in neuronal firing itself is a surprising finding in neuroscience, a simple mathematics further reveals a possible link between the power law and neural code. Namely, we show that *in vivo* cortical neurons try to maximize the firing-rate entropy under joint constraints on the energy consumption and uncertainty of output spike trains. Our results suggest that the brain, which operates under a highly noisy environment and a severe limitation of the energy consumption, may employ a different computational principle from the mutual information maximization in the standard information theory.

## Introduction

The sequences of electrical pulses, or spikes, recorded from *in vivo* cortical neurons are stochastic and highly irregular [1]–[3]. Thus, the brain is a highly noisy information machine. Shannon's information theory and mutual information have often been used to extract the information represented by neural activity [4]–[15]. However, the implications of irregular spike firing for information processing remain elusive. Here, we experimentally and theoretically explore the way cortical neurons represent information about input in the rate of irregular firing by analyzing the spike sequences recorded previously with a juxtacellular electrode in the sensorimotor cortex of behaving rats [16].

We regard a single neuron as a processer that translates the firing rate parameter set by synaptic input into output spike sequence, and ask the relationship between the mathematical principles to describe this translation process and the distribution of inter-spike intervals (ISI). We show that ISIs distribute according to power laws in the majority of pyramidal and fast-spiking inhibitory neurons. The results are unexpected from a hypothesis frequently employed in neuroscience, i.e., the maximization of mutual information (MMI) [8], [17]–[20]. Mutual information represents the amount of information obtained for a probability variable by measuring another variable. A noisy spiking neuron has been shown to maximize mutual information between firing rates and ISIs in given range of firing rate only if it takes discrete values [21]–[23]. However, such a discrete firing-rate distribution should produce an exponential tail in the ISI distribution [23].

In this study, we prove that neurons obey the power-law spike statistics if their noisy activity maximizes the firing-rate entropy under joint constraints on the energy consumption and uncertainty of output spike trains. The maximization of firing-rate entropy (MFE) claims that the distribution of firing rate is determined to maximize the total amount of information represented by firing rate. The MFE is usually accompanied by a constraint that limits the total energy consumption or the range of firing rate [8], [11], [24]–[27]. However, it has been known that the conventional MFE cannot explain the statistical features, such as firing rate distributions, of recorded spike sequences [28], [29]. Several approaches based on MMI between certain internal states and firing rates have been also studied with energy constraint [30]–[33]. Here, we propose a novel hypothesis, i.e., the conditional maximization of firing-rate entropy (CMFE), which adopts an additional constraint on the uncertainty or the variability of output spike trains to account for the power-law statistics. In particular, the constraint is crucial for explaining the wide variation of power-law exponents across the recorded neurons.

## Results

**Figure 1:**
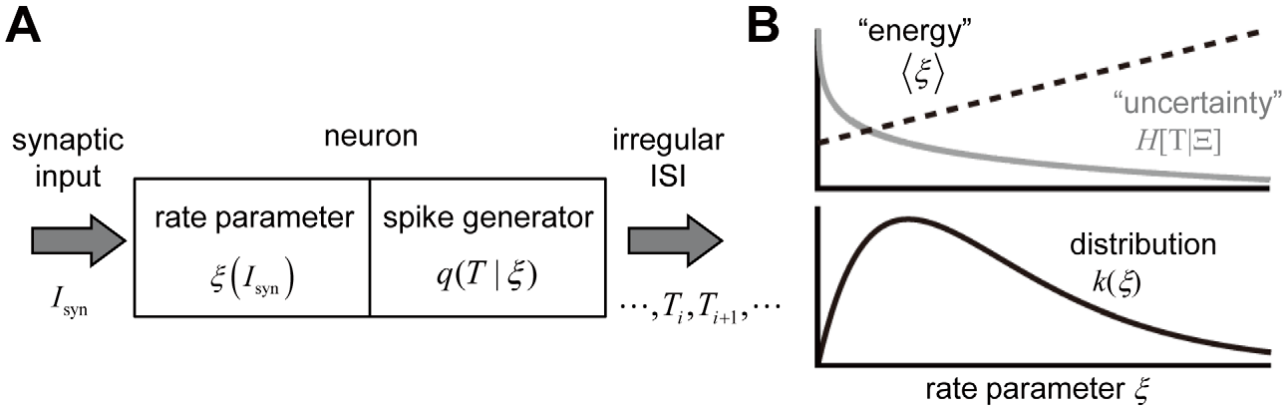
Schematic illustrations of the CMFE and neuronal responses. (**A**) We regard a neuron as a stochastic spike generator to produce a random spike sequence, with ISIs determined by a conditional probability distribution for given rate parameter. The value of *ξ* is determined by synaptic input. (**B**) The CMFE solves a trade-off between the average energy consumption and the uncertainty of output spike trains at given firing rate *ξ*, where the former may be proportional to *ξ* and the latter to −log*ξ* (upper). See Equations 9 and 10. The rate distribution is determined from the balance between the two (lower).

We formulate neuronal firing as a stochastic process to translate the firing rate parameter determined by synaptic input into an irregular sequence of inter-spike intervals. In this view, the rate parameter of a neuron represents an internal state governed by input rather than a neuronal output, and hence is not directly observable (**Figure 1A**). It is quite difficult to estimate time-varying firing rate from a one-shot observation of spike sequence because the maximum likelihood method always biases such estimation. Some studies have proposed an improved estimation method for slowly varying firing rate [34]. However, the method is effective only when the timescale of rate variations does not change with time. Instead of estimating the instantaneous firing rate that can vary rapidly, here we estimate the steady state distribution of firing rate from the inter-spike interval distribution, which is observable. On the basis of this estimation, we will argue that cortical neurons balance the energy consumption and the uncertainty of output spike sequence at given firing rate in maximizing the entropy of firing rate (**Figure 1B**).

### Inter-spike interval distributions of *in vitro* cortical neurons

We summarize results of previous studies of spike statistics of *in vitro* cortical neurons, as they are crucial for the present analysis of irregular firing of *in vivo* cortical neurons. Injecting a fluctuating current with a constant mean and a variance into *in vitro* cortical neurons, we measured irregular sequences of ISI and found that they generally obeyed the gamma distribution (see Figure 2 in Miura et al. [35]). Since the timescale of the fluctuating current (typically, several milliseconds) was much shorter than the typical ISI values (several tens of milliseconds), the fluctuating current was unlikely to modulate the rate of neuronal firing. Thus, a cortical neuron innervated by stationary fluctuating input generates an output spike train of constant firing rate according to the following conditional probability distribution of ISIs [36]–[38]:
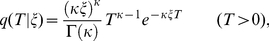
(1)where 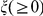 and 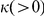 are the rate and shape parameters representing the readiness to fire at a given moment and the (inverse) irregularity of spike firing, respectively.

**Figure 2:**
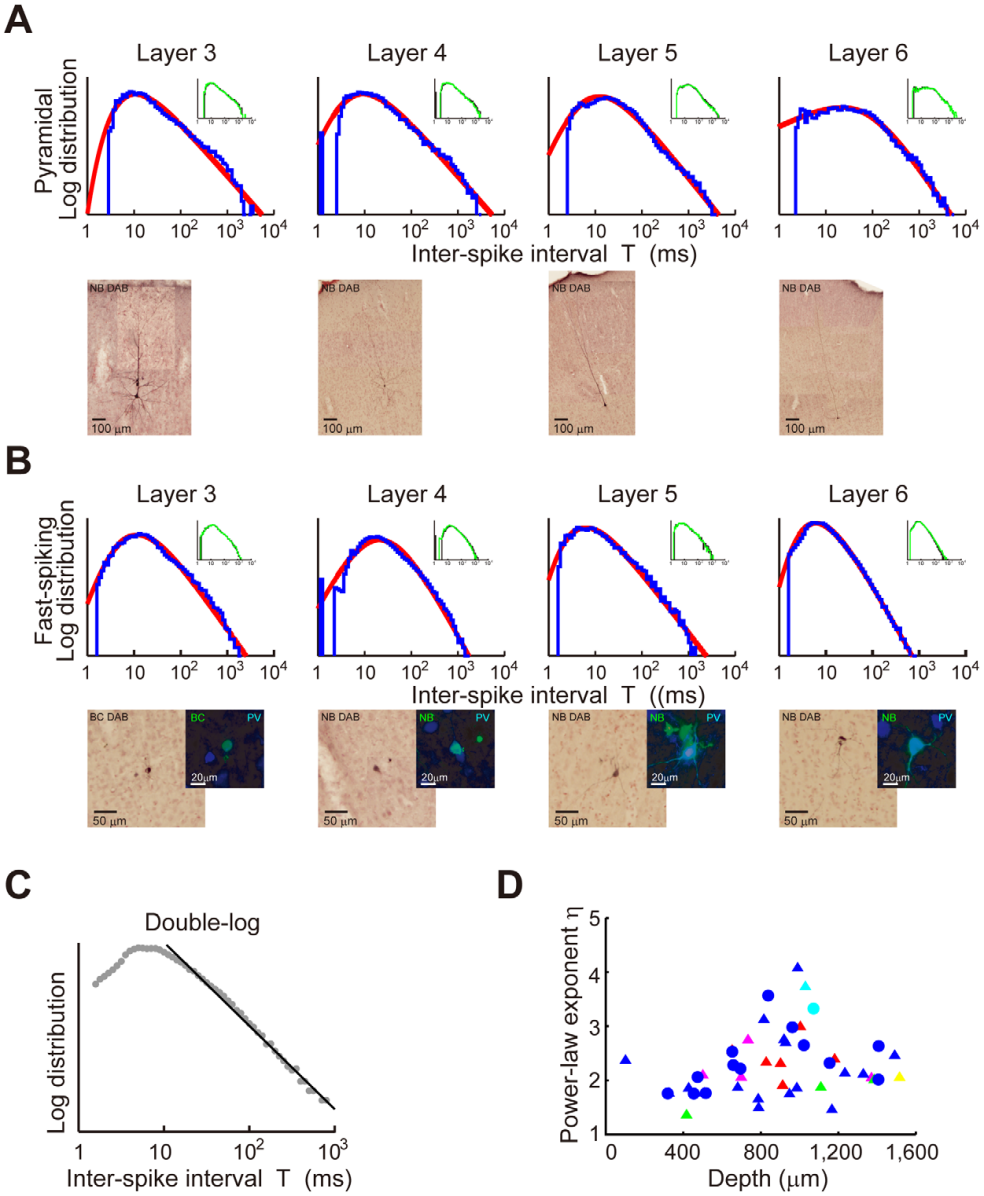
Power-law inter-spike interval histograms of *in vivo* cortical neurons. Juxta-cellular visualization and double-logarithmic plots of the ISI histograms (blue curves) of pyramidal neurons (**A**) and fast-spiking interneurons (**B**) recorded in cortical layers 3, 4, 5 and 6. The plots were fitted by neuron-dependent beta-2 distributions (red curves). The four neurons in (B) expressed parvalbumin (PV), a fast-spiking interneuron specific marker (blue: PV, green: biocytin or Neurobiotin). Inset of each panel represents the ISI distributions constructed from the 1st (black) and 2nd (green) halves of the same spike train. (**C**) Linear regression of the tail of the ISI histogram for one of the 8 neurons shown in (A) (*η* = 2.91, *c.d.* = 0.99). (**D**) The power-law exponents were calculated by linear regression for pyramidal (triangles) and fast-spiking (circles) neurons recorded at various depths of the sensorimotor cortex. Colors indicate the movement components represented by the individual neurons: hold (magenta), pre-movement (green), movement (blue), movement-off (red), post-movement (yellow) and non-related (cyan).

A neuron responding to a stationary input may show constant values of *ξ* and *κ*. For a non-stationary input, the output spike sequence is also non-stationary, and the values of *ξ* and *κ* are difficult to estimate simultaneously and unambiguously. If, however, the irregularity parameter is intrinsic to neurons, we may assume that *κ* changes its value much slower than *ξ* in neurons responding to non-stationary input [34], [35], [39]. In this study, we assume that the value of *κ* would not significantly change in cortical neurons within the time scale of behavior (seconds or longer). The rate parameter will be extrinsic and determined by synaptic input.

### Power-law distributions of inter-spike intervals *in vivo*

We constructed ISI histograms for all the 64 neurons analyzed (**Materials and Methods**). **Figure 2A and B** show examples of the juxtacellular visualization and ISI histograms (blue curves) of 4 pyramidal and 4 fast-spiking neurons in different cortical layers. Note that the plots are in a double-logarithmic scale. All the plots display long tails decaying almost linearly. The result indicates that the ISI distributions obey power laws at long ISIs: 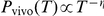, where *T* refers to ISI. To assess how well the power-laws describe the ISI distributions, we analyzed the tails of the ISI histograms of the 64 neurons by linear regression. In more than 70% of the neurons (46/64), the tails were well fitted by linear regression in a double-logarithmic plot, whereas the tails of only 5% neurons (3/64) were well fitted in a semi-logarithmic plot, indicating an exponential slope such as in the gamma distribution (coefficient of determination, *c.d.*>0.95: **Materials and Methods**). **Figure 2C** shows such an example. In only 2 neurons, the tails were better fitted with an exponentially decaying function (data not shown). Neither power-law distributions nor the exponential functions well described the ISI distributions for the remaining neurons.

As shown by the red curves in **Figure 2A and B**, the ISI histograms are well described by the generalized beta distribution of the second kind (beta-2 distribution),
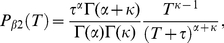
(2)where *Γ*(*x*) (*α*>0, *κ*>0) is the gamma function (**Materials and Methods**). The beta-2 distribution has a power-law tail in the range of large ISIs with exponent *η* = *α*+1. If *κ*>1, the scale parameter *τ* (>0) and the shape parameter *κ* determine the mode *τ*(*κ*−1)/(*α*+1) (i.e., the peak location) and the positive slope ( = *κ*−1) at small ISIs in the double-logarithmic plot of the distribution, respectively. Otherwise, the distribution monotonically decreases for positive *T*. We note that **Equation 2** well fits the ISI histograms even in the range smaller than the modes. The results confirm that the ISIs of irregular firing of cortical neurons obey the beta-2 distribution in behaving rats.

Now, we determined the values of the exponent by a linear fitting of the tails of double logarithmic plots of the ISI distributions (**Figure 2C**). Power laws have been known in the analysis of cortical dynamics. For instance, cortical networks are known to display distinct events of synchronized neuronal firing, with their magnitudes obeying a power-law distribution of an exponent of 3/2 [40]. The unique value of the exponent suggests that spike propagation through cortical networks occurs in critical states of the network dynamics. Unlike these synchronous events, the exponent of the present power law displays a wide range of values (1.37<*η*<4.08) in the ISI histograms of single cortical neurons (**Figure 2D**). As shown later, this result has a significant implication for information-theoretic interpretations of power laws in irregular firing. We further examined whether neurons showing different functional activities have different exponents. The 46 neurons obeying power-law statistics exhibited five different functional activities at all the depths of the recording: hold-related (*n* = 4), pre-movement (*n* = 3), movement (*n* = 30), movement-off (*n* = 6) and post-movement (*n* = 1) activities; some neurons (*n* = 2) showed no obvious link to behavior [16]. We found that the value of the exponent does not show an obvious dependence on the functional category and the neuron type. However, values larger than 2.7 (*n* = 12) were found only at depths between 700 to 1100 µm (*n* = 12; **Figure 2D**).

The beta-2 distributions of ISIs were stationary. To show this, we divided each spike sequence (length 269∼1932 s) into early and late halves containing the same number of spikes. The two ISI histograms constructed separately from the two halves were almost identical (inset in **Figure 2A, B**), meaning that the beta-2 ISI distribution is stationary at least over the time scale of several minutes to several tens of minutes.

### Mixture model for spike generation

In the preceding section, we showed that the irregular ISIs of cortical neurons no longer obey the gamma distribution *in vivo*. At first glance, the results obtained *in vivo* and *in vitro* contradict with one another. We can, however, consistently interpret the results if the value of *ξ* varies for *in vivo* cortical neurons according to a stationary distribution at a timescale longer than the typical ISI. Denoting this distribution by *k*(*ξ*), we describe the distribution of the observed ISIs by the following mixture model:
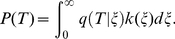
(3)Experimentally, the expression for *k*(*ξ*) should be determined such that *P*(*T*) may coincide with the ISI distribution of *in vivo* cortical neurons. Note that the gamma ISI distribution of *in vitro* cortical neurons is a special case of the mixture model with the rate parameter *ξ* equal to a constant value *R*. In this case, 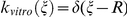 in terms of a delta function and Equation 3 coincides with a gamma function as 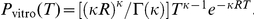


As shown in Equation 2, the ISI distributions observed *in vivo* can be represented by a beta-2 distribution. Then, it is straightforward to derive the expression of *k*(*ξ*) that generates a beta-2 distribution of ISIs. By decomposing *k*(*ξ*) in Equation 3 with Equations 1 and 2, we obtain the following gamma distribution function of the firing rate:
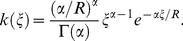
(4)


Equation 4 gives 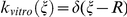 in the limit 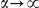, implying that the firing rate of stationary neuronal firing takes a unique value (**Materials and Methods**).

**Figure 3:**
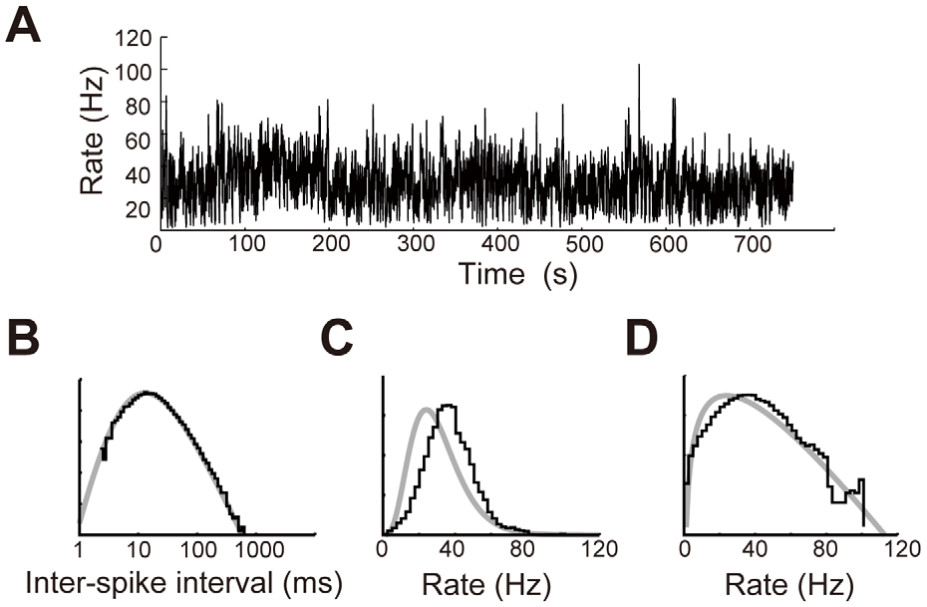
Estimation of the firing rate in cortical neurons. (**A**) The value of *κ* ( = 3.49) was estimated by the method used in Miura et al. (2007) [35] and the instantaneous firing rate was estimated for a cortical neuron according to the method proposed in Koyama and Shinomoto (2005) [34]. (**B**) Double-logarithmic histogram of ISIs constructed for the spike train shown in (A) exhibited a power-law decaying tail (black). The histogram was fitted with a beta-2 distribution (gray). (**C**) Distribution of the estimated instantaneous firing rate (black) was fitted with a gamma distribution (gray). (**D**) The semi-logarithmic plot of the same rate distribution exhibits an exponentially decaying tail (black) characteristic to the gamma distribution (gray).

We examined whether *k*(*ξ*) actually obeys a gamma distribution. To this end, we estimated the instantaneous values of firing rate for experimentally recorded spike trains by using a previously proposed method [34] and constructed the distributions of these values. **Figure 3** displays typical examples of thus constructed *k*(*ξ*). As predicted theoretically, *k*(*ξ*) was well described with a gamma distribution. Below, we use Equation 4 for exploring the coding scheme of cortical neurons.

### Maximization of firing-rate entropy hypothesis

The distribution function of firing rate is tightly linked with the way neurons encode information into the firing rate of spike trains, and several hypotheses have been proposed for the neural information coding. A well-studied hypothesis about *k*(*ξ*) is the MFE hypothesis, which claims that *k*(*ξ*) is determined to maximize the entropy of a time-varying firing rate,
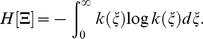
(5)The entropy is a functional of *k*(*ξ*) and indicates the amount of total information represented by the rate distribution. If we maximize *H*[Ξ] by taking its functional derivative with respect to *k*(*ξ*), we obtain a uniform distribution for *k*(*ξ*). This means that the firing rate takes all possible values (below a certain maximum value) with an equal probability, which is biologically unrealistic. Therefore, to obtain a biologically relevant structure for *k*(ξ), we have to maximize *H*[Ξ] under certain constraints. The MFE hypothesis often adopts a constraint on the total energy consumption of neuronal firing. The total energy cost of spike generation is approximately proportional to the number of spikes if the energy cost per spike depends weakly on the firing rate [8], [24]–[26]. Therefore, we may seek the firing rate distribution that can maximize *H*[Ξ] under the following constraint on the average firing rate:
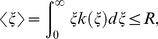
(6)where *R* is a constant.

The solution to this maximization problem is an exponential distribution function:
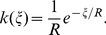
(7)Namely, we solve the maximization problem by functional variation of Equation 5 with Lagrange multipliers *λ*_0_ and 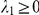 [41]:
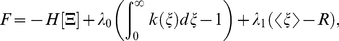
(8)where the second and third terms impose the normalization condition on *k*(*ξ*) and the constraint shown in Equation 6, respectively. We note the sum of the first and third terms is analogous to the so-called free energy in the thermodynamics. At the solution to the maximization problem, the functional derivative of *F* with respect to *k*(*ξ*) should vanish: *δF*/*δk* = 0. From this stationary condition and Equation 8, we can derive a condition on *k*(*ξ*) as
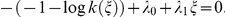
Solving this equation yields the solution of the MFE given in Equation 7. Note that Equation 4 coincides with Equation 7 in a special case of *α* = 1.

Using Equations 1, 3 and 7, we obtain the ISI distribution for the neuronal firing that obeys the MFE hypothesis as
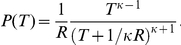
This distribution has a power-law tail 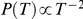 in the range of ISIs much longer than the mode, 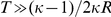. In the range of short ISIs, the distribution behaves as 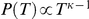. Thus, the MFE hypothesis gives power-law ISI distributions that resemble those observed *in vivo* in cortical neurons. Nevertheless, this hypothesis is not supported by our results since it allows the exponent to take only a single value (*η* = 2), whereas the experimentally observed values are widely distributed around this theoretical value (**Figure 2D**).

### Maximization of mutual information hypothesis

Another well-studied hypothesis is the maximization of mutual information (MMI). Mutual information tells us how much information about the rate parameter can be read out from the spike sequences. In the MMI hypothesis, neurons would choose such a rate distribution *k*(*ξ*) that maximizes the mutual information between the value of *ξ* and the ISIs of spike sequence. In order to transmit a large amount of information, the system needs to have a number of (or densely distributed) communication channels. However, as the number of channels is increased, to secure distinct signal levels for different channels becomes difficult and the chance of noise interference may increase. Therefore, an effective solution to this trade-off is necessary for MMI. Under constraint on the power consumption, Gaussian distributed analog signals are widely known to achieve MMI for additive Gaussian white noise channel [4], [9], [26]. In many other communication channels, however, MMI is only achievable with discrete signals [21]–[23]. For example, Shamai [21] showed that the firing-rate distribution for MMI is discrete in the case of Poisson channels of spike intervals when the average firing rate is constrained. In the present gamma-distribution spike-interval channels, MMI was proved for a discrete firing-rate distribution in the presence of the upper and lower bounds for the firing rate [23].

Thus, noisy spiking neurons can achieve the maximum information transmission only when the distribution of firing rate is discrete, including a binary case where the firing rate can take only two values, say, low and high firing rates *R*_low_ and *R*_high_: 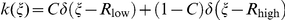, where 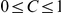 is the ratio of the low firing rate to the high firing rate. Therefore, if a cortical neuron obeyed the MMI principle, its ISI distribution would be a superposition of a finite number of gamma distributions,
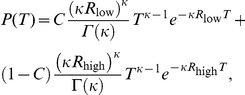
which implies that the tail of the ISI histogram should exhibit exponential decay. Thus, our experimental results suggest that MMI hypothesis is unlikely to hold for cortical neurons *in vivo*.

### Conditional maximization of firing-rate entropy hypothesis

Now we may ask what kind of hypothesis leads to the firing rate distribution derived experimentally in Equation 4. Does such a hypothesis exist? To answer the questions, we introduce an additional constraint in the MFE hypothesis to restrict the uncertainty (noise) of the spike generation by *q*(*T*|*ξ*). We define the conditional response entropy *H*[T|Ξ] (or neuronal noise according to Borst and Theunissen, 1999 [9]) associated with irregular neuronal firing as
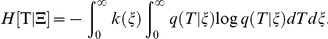
The conditional response entropy implies the average uncertainty of the ISIs generated by the stochastic spike generator *q*(*T*|*ξ*) at given firing rate *ξ*. The smaller the conditional response entropy is, the more reliable the spike generation is [3]. We propose to impose a constraint on *H*[T|Ξ], in addition to the constraint on the energy consumption, in maximizing the amount of total information represented by the distributed firing rates. Thus, our task is to find the expression of *k*(*ξ*) that maximizes *H*[Ξ] under the two constraints, 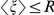 and 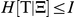, where *R* and *I* are some constants. The values of these constants may vary from neuron to neuron. The second constraint says that there is an upper bound for the unreliability of spike generation. Since *H*[T|Ξ] is a functional of *k*(*ξ*), the additional constraint will further restrict the range of firing rates to be used for information transmission.

We can solve the above maximization problem by functional variation of Equation 5 with three Lagrange multipliers 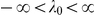 and 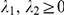:
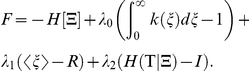
Then, solving the stationary condition *δF*/*δk* = 0, we obtain
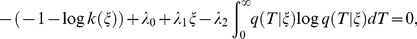
(9)where the last term is the entropy of the stochastic spike generator *q*(*T*|*ξ*). Below, we solve Equation 9 to obtain the expression for *k*(*ξ*).

We can find a solution to Equation 9 if the spike generator belongs to a family of “scale-invariant” probability distributions satisfying
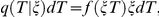
where *f*(*x*) (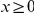) is a probability distribution function with mean unity [42]. This family gives well-defined firing rate and inter-spike intervals, such that the average ISI (the average of *T*) coincides with the inverse of *ξ*, and involves a wide class of distributions including exponential (Poisson process), gamma, log-normal, inverse-Gaussian and Weibull distributions [43]. For this family, we can rewrite the last term in Equation 9 as
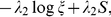
(10)where 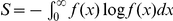 is the rate-independent entropy of *f*(*x*). For the gamma distribution 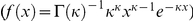, we obtain 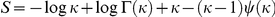 where 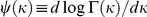 is the digamma function. Note that *S* only depends on the shape parameter *κ* of *q*(*T*|*ξ*). Now we can solve Equation 9 to show that *k*(*ξ*) is expressed as a gamma function: 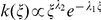. By determining the Lagrange multipliers and comparing the resultant expression of *k*(*ξ*) with Equation 9, we find relationships between the parameters: the solution to the maximization problem is obtained by solving 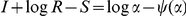 if *α*>1, or otherwise *α* = 1.

Thus, the gamma distribution of the instantaneous firing rate is not only consistent with the power-law ISI distributions of *in vivo* cortical neurons, but also maximizes the total information amount represented by the firing rate with upper bounds for the mean firing rate and conditional response entropy of ISIs. We referred to this extended MFE hypothesis as the conditional maximization of firing-rate entropy (CMFE). The CMFE hypothesis implies that cortical neurons try to maximize the information amount, while suppressing the energy cost and influences of noise on spike generation.

### Diversity of spiking characteristics of individual neurons

**Figure 4:**
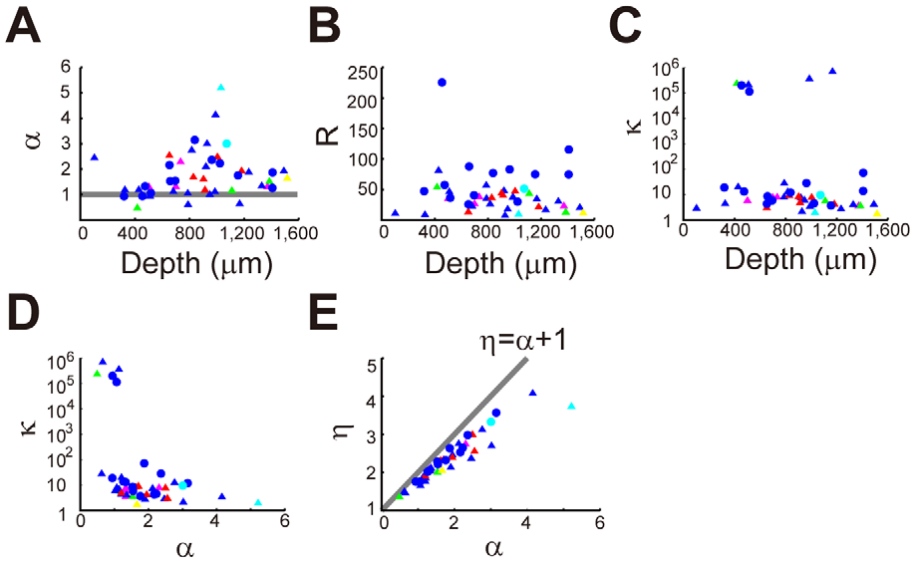
Neuron-dependence of parameter values of the double gamma process. (**A**)–(**C**) Optimal values of beta-2 fitting parameters *α*, *R* and *κ* for pyramidal (triangles) and fast-spiking (circles) neurons recorded at various depths of the cortex. As in **Figure 2**, the color code indicates the motor components represented by the individual neurons. The values were determined by nonlinear least square method (**Materials and Methods**). (**D**) The relationship between *α* and *κ* values over the recorded neural population. (**E**) The relationship between *α* and *η* values. The latter was determined by linear regression of the power-law tails of ISI distributions (see **Figure 2C**).

If the CMFE hypothesis is correct, the ISI distributions characterized by three parameters *α*, *κ* and *τ* (Equation 2) should be decomposed into two gamma distributions — *k*(*ξ*) with the mean rate *R* and regularity parameter *α* (Equation 4) and *q*(*T*|*ξ*) with the regularity parameter *κ* (Equation 1), where *R* = *α/τκ* should hold. To see the variations in the values of *κ*, *α* and *R* over a population of cortical neurons, we plotted these values for all the recorded neurons. The values were determined by the least-square fitting of double logarithmic plots of the ISI distributions of cortical neurons with the beta-2 distribution given in Equation 2 (**Materials and Methods**). The obtained parameter values were significantly different from neuron to neuron (**Figure 4A–C**). The results show that the CMFE is valid in most of the neurons yielding 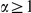 (six neurons yielded *α*<1, and three neurons among them showed *α*-*v*alues significantly smaller than 1: see **Figure 4A**). Neurons with relatively large value of *α* were found only at depths between 700 to 1100 µm, implying that the temporal variability of the rate parameter was small in these neurons. The values of *R* and *κ* showed no remarkable differences between the superficial and deep layers (**Figure 4B, C**). **Figure 4D** displays the values of *κ* and *α* for all the recorded neurons. We only found neurons that have large *κ* and small *α*, small *κ* and large *α*, or small *κ* and small *α*. Very large *κ* values of the first group, however, could be partly due to the fitting errors. The present data contained no neurons with large values of *κ* and *α*. We previously calculated the values of the power-law exponent *η* from the linear slopes of the double logarithmic plots (**Figure 2D**). If we compare the results with those shown in **Figure 4A**, the expected relationship *η*∼*α*+1 holds approximately for the present data, although systematic deviations arose from errors in the fitting of the distributions at small ISIs (**Figure 4E**).

**Figure 5:**
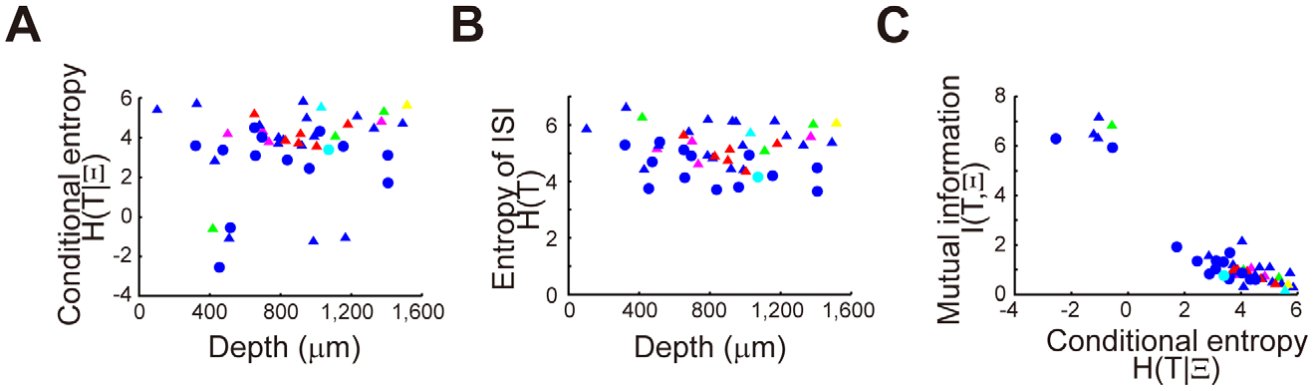
Information measures for cortical neurons. (**A, B**) The conditional response entropy and entropy of ISI distribuions, respectively, for pyramidal (triangles) and fast-spiking (circles) neurons recorded at various depths of the cortex. Colors indicate the motor component represented by each neuron (see the legend of **Figure 2D**). (**C**) Correlations between the conditional response entropy and mutual information for the individual neurons.

The two parameters *α* and *κ* characterize the different aspects of irregular neuronal firing: κ^−1^ measures the irregularity of the output spike train at a given rate parameter, while *α* ^−1^ measures the degree of the rate modulation. It is therefore intriguing to see the overall irregularity represented by the individual neurons. To this end, we calculated the conditional response entropy *H*[T|Ξ] and the entropy *H*[T] of output spike trains as overall irregularity measures. The conditional response entropy takes widely distributed values in both superficial and deep layers (**Figure 5A**), whereas the entropy *H*[T] changes only moderately among different neurons (3.7<*H*[T]<6.6: **Figure 5B**). Thus, the mutual information *I*[T,Ξ] = *H*[T]−*H*[T|Ξ] between the rate parameter and ISI is negatively correlated with the conditional response entropy *H*[T|Ξ] (**Figure 5C**). This result implies that different neurons represent a nearly equal variety of output spike trains *H*[T], regardless of the fidelity of intrinsic spike generation *H*[T|Ξ]. Indeed, the constant output entropy *H*[T] reflects the negatively correlated distribution of *κ* and *α* values over the neuron ensemble (**Figure 4D**).

## Discussion

Whether irregular firing of cortical neurons is crucial for the information representation in the brain remains unclear. To achieve an insight into this issue, here, we have investigated the statistics of spike trains recorded from the sensorimotor cortex of behaving rats. We have shown that ISI distributions display heavy tails decaying with power laws in more than 70% of the neurons recorded. We have explored a possible link of the power-law spiking statistics with neural code, and have shown that such statistics is consistent with the conditional maximization of the firing-rate entropy under constraints on the energy consumption and uncertainty of output spike trains. The results imply that the maximization of mutual information (MMI) does not necessarily hold for information processing by cortical neurons. Rather, the solution to this conditional maximization problem specifies a distribution function of firing rate representing the distributed communication bands of individual neurons.

### Conditional maximization of firing rate entropy

We may regard a neuron as a processor to translate synaptic input into a spike train, where the instantaneous value of the rate parameter is determined by synaptic input to each neuron. The rate parameter should coincide with the average firing rate of the neuron for stationary synaptic input, whereas it may vary dynamically and fluctuate in time for non-stationary synaptic input. We have shown that the rate parameter obeys a gamma distribution in spike trains of sensorimotor cortical neurons. Moreover, we have theoretically proved that this distribution follows from a general hypothesis for the cost-information trade-off in irregular neuronal firing, i.e., the CMFE (the conditional maximization of the firing-rate entropy), which claims to maximize the entropy of firing rate under constraints on the average firing rate and response entropy of inter-spike intervals for given firing rate. Here, the firing-rate entropy means the variety of firing rates available for neuronal communication.

Now we consider the implications of the CMFE for irregular spike generation. The CMFE hypothesis is an extension of the “maximum entropy” with additional constraints on the conditional response entropy *H*[T|Ξ]:
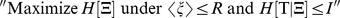
We may rewrite the above expression into the following minimization problem:
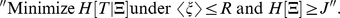
Here, the two expressions give mathematically identical solutions if the equalities hold for the constraints. In the new expression, *H*[T|Ξ] is minimized under constraints on the average firing rate and the entropy of firing rate. The conditional response entropy represents the uncertainty left for a variable (ISI in the present case) after a measurement of the other (firing rate). The average energy consumed by a neuron may increase proportionally with firing rate. However, the conditional entropy will be increased for output spike trains if neurons use lower firing rate more frequently. Thus, our results imply that neurons in the sensorimotor cortex minimize the average uncertainty of output spike sequences, while balancing the tradeoff between the energy consumption and the total information amount *H*[Ξ] obtained from the firing rate [24], [25], [27], [30]–[33]. In other words, if we may regard 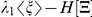 as the “free energy” of neuronal firing, the CMFE solves a trade-off between the free-energy and conditional response entropy [24].

Our finding has the following implications for cortical information processing. In general, noise may differentially affect the reliability of signal transmissions at different firing rates since the timing jitter of spikes can grow larger at a lower firing rate. The CMFE implies that neuronal networks temporally distribute the use of different frequency bands to minimize the average entropy of spike firing by a certain single-cell or network-level mechanism. Namely, the average entropy of spike trains is minimized for given mean and entropy of firing rate when it obeys a gamma distribution: 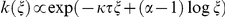. In fact, the particular distribution solves the tradeoff between the energy cost (*ξ*) and the entropy (−log *ξ*) of spike firing (**Figure 1B**).

### Comparison between CMFE and MMI

A widely adopted hypothesis is that neurons maximize mutual information between input and output. MMI also provides a powerful tool to decipher neural code. However, MMI did not explain information transmission in the cricket auditory neuron [44]. Furthermore, MMI achieves the channel capacity, which represents a theoretical upper bound for transmittable mutual information [4], [8], , only if inputs are restricted within a narrow range, typically discrete values [21]–[23], determined by physical properties of the communication channel and noise.

It is worthwhile to compare the CMFE with MMI. We may define the mutual information between firing rate and inter-spike intervals as
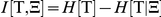
in terms of *H*[T|Ξ] and the entropy of inter-spike intervals *H*[T]. Mathematically, we can obtain the solutions to MMI and CMFE by solving the equations 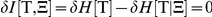 or 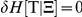, respectively. If the average entropy of ISIs is fixed, i.e., 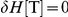, the two equations are equivalent, so the CMFE and MMI yield the same solution to the rate distribution. However, the condition does not necessarily hold for CMFE. Actually, the gamma distribution of firing rate does not satisfy it: 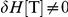.

The importance of firing rate in information coding by noisy cortical networks has been demonstrated repeatedly [2], [11], [18], [24], [45]. Our finding of the CMFE further demonstrates the way neurons translate information on firing rate into irregular spike trains based on experimental data and mathematical analysis. Our study explicitly shows a mathematical principle for neural information coding alternative to the MMI. In fact, the brain works under a severe limitation of the energy consumption, and its highly noisy computation may require a different principle. Our results shed light on the principle of neural information coding and may uncover an essential difference between the brain and artificial machines that often rely on the MMI.

### “Super-statistics” in cortical neural processing

Our findings imply that the ISIs of *in vivo* cortical neurons significantly vary over time without a typical time scale. Since the irregular firing of cortical neurons intrinsically obeys the gamma process, as shown for balanced synaptic input [35], the core mechanism of the CMFE is that which governs the temporal distribution of firing rate in individual neurons. Though we speculate that a certain network-level mechanism underlie this process, the mechanism of the scale-free cortical dynamics remains to be clarified. It also remains open for further studies whether an alternative explanation of the power-law spike statistics exists. We may, for example, test whether synchronization of input spike sequence [3], [46], the heterogeneity of neuronal properties [47], [48] or a specific design principle of neuronal networks [49] can generates the power laws.

We have shown that the power-law ISI distribution of irregular neuronal firing *in vivo* is described by double gamma distributions: a first gamma distribution describes fast spike generation at a fixed rate parameter, and a second one characterizes slower temporal fluctuations in the rate parameter. Such a mixture model, generally known as “super-statistics”, describes a superposition of multiple differing statistical models with hierarchically different time scales and has been verified in various phenomena. For instance, the examples include the distribution of traded volumes in financial markets [50] and the energy density distribution of non-equilibrium states of turbulence [51], [52]. Here, we have verified the super-statistics on sensorimotor cortical activity and derived the CMFE for the neuronal information processing obeying this statistics. A similar power-law statistics was reported previously in cat primary visual cortex and macaque inferior temporal (IT) areas [25], suggesting that the CMFE is also valid for sensory information processing. On the other side, there are some other studies suggested different statistical distributions [37], [53]–[55]. It seems worthwhile to make a detailed comparison between their data and ours because the recording methods, recorded animals, areas and states were different. Here, juxtacellular recordings enabled us not only to identify the morphology and location of recorded neurons [56], [57], but also to record spike sequences accurately enough to identify the power-law tails extending over more than two orders of ISI. We should, however, point out the possibility that the juxtacellular recordings may modify the cellular firing properties due to the very short distance between the glass tip of the recording electrode and the cell membrane.

In summary, the CMFE posits that cortical neurons maximize the total information amount represented by firing rate, simultaneously solving the trade-off between noise and the energy consumption. To our knowledge, our study is the first that proposes the essential relevance of the CMFE to neural code. Since the CMFE has rarely been studied in artificial machines, its implications for information processing should be further clarified.

## Materials and Methods

### Ethics statement

All experiments were performed in accordance with animal protocols approved by the Experimental Animal Committee of the RIKEN Institute.

### Juxtacellular recording from behaving rats

To obtain a sufficient number of spike events for the present analysis, we reanalyzed the raw electrophysiological data recorded previously in Isomura et al. (2009) [16]. Here, we briefly summarize the experimental procedure since the details are found in the paper. Adult male Long-Evans rats (150–250 g; Japan SLC) were trained to perform self-paced right forelimb movements (sequence of push-, hold- and pull-movements of a lever) after we surgically attached a lightweight, custom-made sliding head-attachment to the skull of the rats. Then we recorded the activity of 87 neurons in the layers 2–6 of the sensorimotor cortex juxtacellularly from the head-restrained rats performing the behavioural task, in which the rats voluntarily repeated a sequence of push-hold-pull of a lever. The rats were rewarded if they pulled a lever after they hold it at a push position for more than 1 second. After recording of task-related spike activity of a single neuron, biocytin or Neurobiotin was electroporated from a glass electrode into the recorded neuron with positive current pulses to obtain the morphological information and cortical position of the neuron. Biotin/Neurobiotin-loaded neurons were visualized with streptavidin-AlexaFluor488 (Molecular Probes, Inc.) in combination with double-immunostaining for parvalbumin and calretinin. The electrode depth was also used to estimate the position of the recorded neurons. Juxtacellular recording allowed us to record precise spike sequences from morphologically identified neurons. We selected 64 sequences (neurons) that contained more than 2000 spikes for the present analysis (sequence length: 269 s∼1932 s; firing frequency: 2.5 Hz∼50.9 Hz). The remaining 23 neurons were not included, as they did not contain sufficiently many spikes for statistically meaningful analysis of long-tailed distributions. Thirty-one of 64 neurons were successfully identified as pyramidal neuron (*n* = 22) and interneuron (*n* = 9) by DAB-Nickel staining. Eight of the nine interneurons were parvalbumin positive, which is a chemical marker of fast-spiking interneuron [58], [59], and one interneuron was parvalbumin-negative (and also calretinin-negative). The unlabeled 33 neurons were categorized by the mean firing rate and the averaged spike width according to the criteria described previously [16]: neurons with the average firing rate of less than 30 Hz and the average spike width of more than 0.2 ms were classified as putative pyramidal cells; the others as putative fast-spiking interneurons. In total, we obtained 47 (identified and putative) pyramidal and 17 fast-spiking interneurons for the present analysis.

### Construction of power-law ISI histograms

We calculated inter-spike intervals *T_j_* = *t_j_*_+1_−*t_j_* of the spike sequences {*t_j_*} recorded from cortical neurons and constructed histograms for {*T_j_*}. Because events with longer inter-spike intervals rarely occur, the bin counts show larger fluctuations at longer ISIs than at shorter ones. To solve this problem, we used a logarithmic binning [60] by setting the width of the *j*th bin for ISI values in [10^(*j*−1)/*M*^ ms, 10*^j/M^* ms] to *ΔT_j_* = (1−10^−1/*M*^)10*^j/M^* ms (*j* = 1, 2, …, 4*M*). We normalized the event count *h_j_* in the *j*th bin by *ΔT_j_* to obtain the probability that an inter-spike interval falls into the bin. We chose the total number of bins as 4*M* = 80 (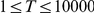 ms) in the present study.

In **Figure 2C**, we applied linear regression to the tail of each ISI histogram in the range of ISIs that are two-fold larger than the mode of the histogram. We defined the coefficient of determination (*c.d.* [61]) as
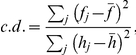
where “predicted value” *f_j_* is given as *f_j_* = −*η*log*T_j_*+*δ* in a double-logarithmic plot and *f_j_* = −*ηT_j_*+*δ* in a semi-logarithmic plot in terms of regression parameters, and 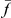 and 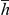 represent the average values. The *c.d.* takes values between 0 and 1. If all the bin counts coincide with the predicted values, *h_j_* = *f_j_*, and the *c.d.* becomes unity.

### Generalized beta distribution of the second kind

To describe the tails of ISI distributions, we introduced a family of power-law distributions called “the generalized beta distribution of the second kind” or “beta-2 distribution” in Equation 2 [43], [62]. The beta-2 distribution has a power-law tail 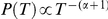 in the range of ISIs longer than the mode. In the range of short ISIs, the beta-2 distribution behaves as 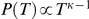. The beta-2 distribution has *n*th moments only for *n*<*α*. If *α*>2, the average and coefficient of variance (*CV*) of the beta-2 distribution are well defined as *α*/((*α*−1)*R*) and 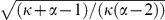, respectively.

We used a nonlinear least mean square method (Marquardt-Levenberg method [63], [64] to estimate the values of *κ*, *α* and *τ* that best fit each ISI histogram. This method iteratively renews the model parameters to minimize the discrepancy between the data and the model. The curve fitting was performed on the logarithm of the beta-2 distribution, 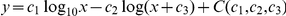, where *κ* = *c*_1_+1, *α* = *c*_2_−*c*_1_−1, *τ* = *c*_3_ and *C*(*c*_1_,*c*_2_,*c*_3_) is a normalization constant.

### Firing rate distributions for power-law-distributed ISIs

We show how to derive the distribution *k*(*ξ*) of the rate parameter presented in Equation 4 from the stochastic spike generator *q*(*T*|*ξ*) and the ISI distribution represented by the beta-2 distribution given in Equation 2. Straightforwardly, we may equate Equation 2 with the following expression:
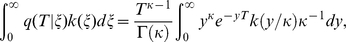
where 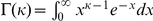 is the gamma function. We have changed the integration variable as *y* = *κξ* after replacing *q*(*T*|*ξ*) with the gamma distribution known from *in vitro* recordings: 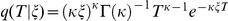 (see Equation 1). Then, taking an inverse Laplace transform and noting the definition of the beta function, we can represent the unknown rate distribution as
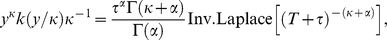
which is calculated as in Equation 4 if we set the parameters as 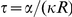.

### Large-α limit of the gamma distribution

We show that the gamma distribution shown in Equation 4 approaches a delta function as α goes to infinity. Explicitly, by taking the logarithm of the gamma distribution 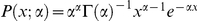 and using an asymptotic expansion of the gamma function
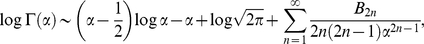
(*B_2n_* is the Bernoulli number), we obtain
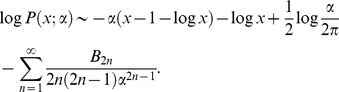
For any positive value of *x*, 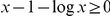. Therefore, as *α* goes to infinity, the negative leading term of order *α* vanishes and 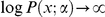 if and only if *x* is unity (because the next leading term of order log*α* diverges). This means that 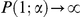 as 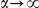. If *x* is not unity, 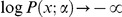 and 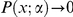 as 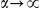. Since 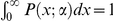, we obtain 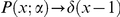 in the limit 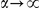.
